# Activation of Invariant NKT Cells with Glycolipid Ligand α-Galactosylceramide Ameliorates Glucose-6-Phosphate Isomerase Peptide-Induced Arthritis

**DOI:** 10.1371/journal.pone.0051215

**Published:** 2012-12-12

**Authors:** Masanobu Horikoshi, Daisuke Goto, Seiji Segawa, Yohei Yoshiga, Keiichi Iwanami, Asuka Inoue, Yuki Tanaka, Isao Matsumoto, Takayuki Sumida

**Affiliations:** 1 Department of Internal Medicine, Faculty of Medicine, University of Tsukuba, Tsukuba, Japan; 2 Department of Internal Medicine, University of New Mexico, Albuquerque, New Mexico, United States of America; University Hospital Jena, Germany

## Abstract

**Objective:**

Invariant natural killer T (iNKT) cells regulate collagen-induced arthritis (CIA) when activated by their potent glycolipid ligand, alpha-galactosylceramide (α-GalCer). Glucose-6-phosphate isomerase (GPI)-induced arthritis is a closer model of human rheumatoid arthritis based on its association with CD4+ T cells and cytokines such as TNF-α and IL-6 than CIA. Dominant T cell epitope peptide of GPI (GPI325-339) can induce arthritis similar to GPI-induced arthritis. In this study, we investigated the roles of activation of iNKT cells by α-GalCer in GPI peptide-induced arthritis.

**Methods:**

Arthritis was induced in susceptible DBA1 mice with GPI peptide and its severity was assessed clinically. The arthritic mice were treated with either the vehicle (DMSO) or α-GalCer. iNKT cells were detected in draining lymph nodes (dLNs) by flow cytometry, while serum anti-GPI antibody levels were measured by enzyme-linked immunosorbent assay. To evaluate GPI peptide-specific cytokine production from CD4+ T cells, immunized mice were euthanized and dLN CD4+ cells were re-stimulated by GPI-peptide in the presence of antigen-presenting cells.

**Results:**

α-GalCer induced iNKT cell expansion in dLNs and significantly decreased the severity of GPI peptide-induced arthritis. In α-GalCer-treated mice, anti-GPI antibody production (total IgG, IgG1, IgG2b) and IL-17, IFN-γ, IL-2, and TNF-α produced by GPI peptide-specific T cells were significantly suppressed at day 10. Moreover, GPI-reactive T cells from mice immunized with GPI and α-GalCer did not generate any cytokines even when these cells were co-cultured with APC from mice immunized with GPI alone. *In vitro* depletion of iNKT cells did not alter the suppressive effect of α-GalCer on CD4+ T cells.

**Conclusion:**

α-GalCer significantly suppressed GPI peptide-induced arthritis through the suppression of GPI-specific CD4+ T cells.

## Introduction

Rheumatoid arthritis (RA) is a chronic polyarthritic inflammatory disease of the synovial membranes. Although the etiology of RA is considered to be an autoimmune reactivity to certain self antigens, the exact mechanism remains obscure. Accumulating evidence suggests that CD4+ helper T cells play an important role in the pathogenesis of RA [1]. Invariant natural killer T (iNKT) cells are a unique subset of T cells that co-expresses NK markers, such as NK1.1 and a highly restricted TCR repertoire, composed of a single invariant α chain (Vα14-Jα18 in mice and Vα24-Jα18 in humans), together with a limited TCR Vβ repertoire. When iNKT cells recognize glycolipid ligands presented by the class I major histocompatibility complex (MHC)–like molecule CD1d on antigen presenting cells (APCs), they rapidly respond by producing large amounts of Th1, Th2, and Th17 cytokines [2]–[4]. The potent exogenous ligand of iNKT cells, α-galactosylceramide (α-GalCer), has been used for the treatment of several types of murine autoimmune models such as type 1 diabetes, experimental autoimmune encephalomyelitis (EAE), and collagen-induced arthritis (CIA) [5]–[9]. The effects of α-GalCer on these autoimmune diseases are considered to be mediated through the induction of antigen-specific IL-10 production [8], [10], foxp3+ regulatory T (Treg) cells [11], [12], and regulatory dendritic cells [13]. However, the role of α-GalCer in various autoimmune diseases, including RA, remains to be elucidated.

Glucose-6-phosphate isomerase (GPI) is an arthritogenic autoantigen identified in KxB/N mice [14]. GPI can provoke arthritis in susceptible DBA1 mice [15]. GPI-induced arthritis is considered to be a closer model of human RA than CIA with regard to its dependency on CD4+ T cells and response to biological agents, such as anti-TNF-α and anti-IL-6 receptor antibody [16], [17]. GPI-induced arthritis is characterized by early-onset of clinical signs of arthritis, which usually develop around day 8, with an early peak on day14. We and Bruns et al. demonstrated previously that the major epitope of T cells in GPI-induced arthritis is human GPI325–339, and that immunization with the 15-mer peptide can provoke GPI peptide-induced arthritis, which is similar to GPI-induced arthritis [18], [19].

The present study is an extension to our previous studies on the role of α-GalCer in GPI peptide-induced arthritis. The results showed that α-GalCer activated iNKT cells and provided protection against GPI peptide-induced arthritis. The results also showed that α-GalCer suppressed GPI-specific CD4+ Th1 and Th17 cell response and anti-GPI autoantibody production by B cells. Thus, in the T cell dependent arthritis model, α-GalCer seems to suppress arthritis through antigen-specific regulation, suggesting a potentially useful therapeutic strategy against human RA through iNKT cell ligands.

**Figure 1:**
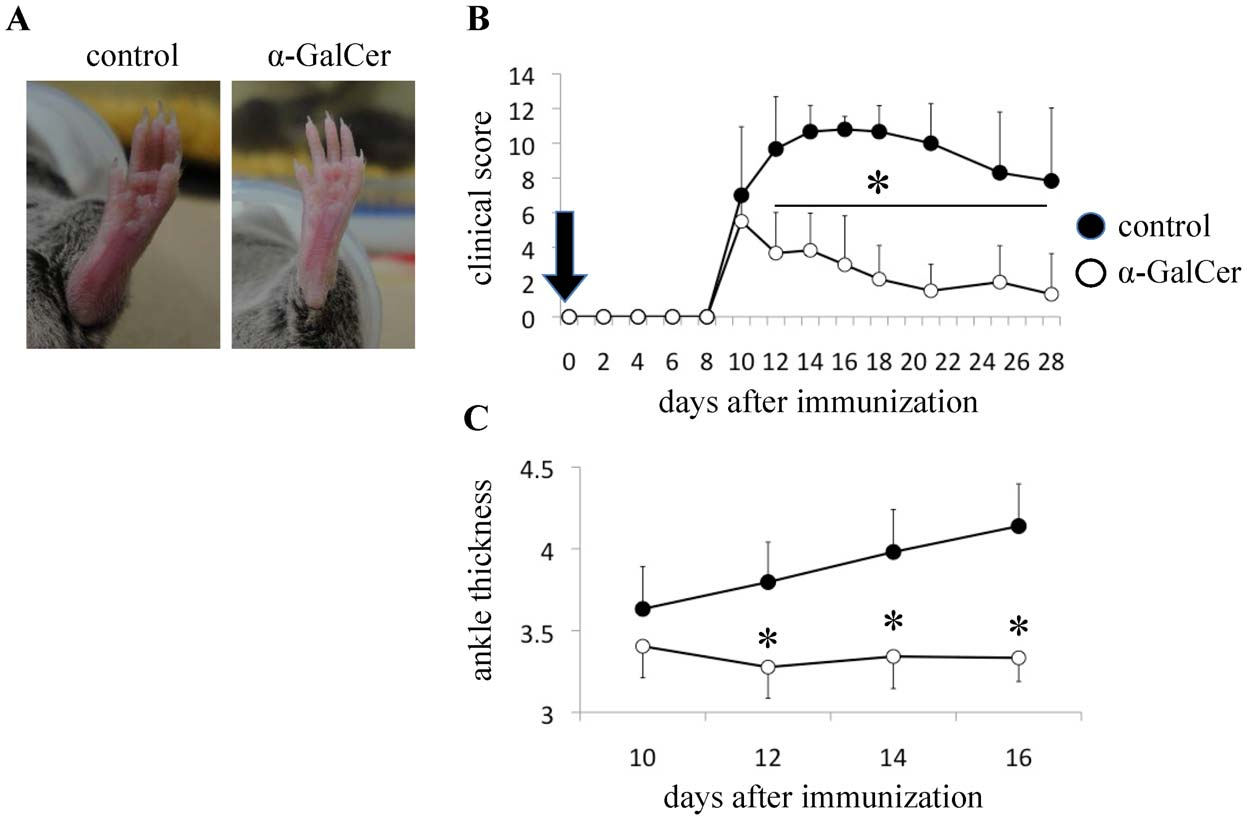
α-GalCer significantly suppressed the severity of GPI peptide-induced arthritis. DBA1 mice were immunized with GPI peptide and then treated with either DMSO (as a vehicle control) or α-GalCer followed by clinical assessment of arthritis. (n = 5, data shown are representative results of three experiments). (**A**) Severe swelling of ankle joints of control mice and markedly improved swelling of the ankle joints of α-GalCer-treated mice. (**B**) Clinical score, and (**C**) Ankle thickness. The latter represented the severity of arthritis. Arrow indicates α-GalCer administration. Data are mean±SD. *p<0.01, by Man-Whitney test.

## Materials and Methods

### Mice

Male DBA/1J mice were purchased from Charles River Japan (Tokyo, Japan). The animals were kept under specific pathogen-free conditions in our animal facility and studied at 7–10 weeks of age. The Institutional Animal Care and Use Committee of the University of Tsukuba approved all the experimental protocols (Permit number: 2010–116 and 2011–119). All the surgery was performed under isoflurane anesthesia, and all the efforts were made to minimize suffering.

**Figure 2:**
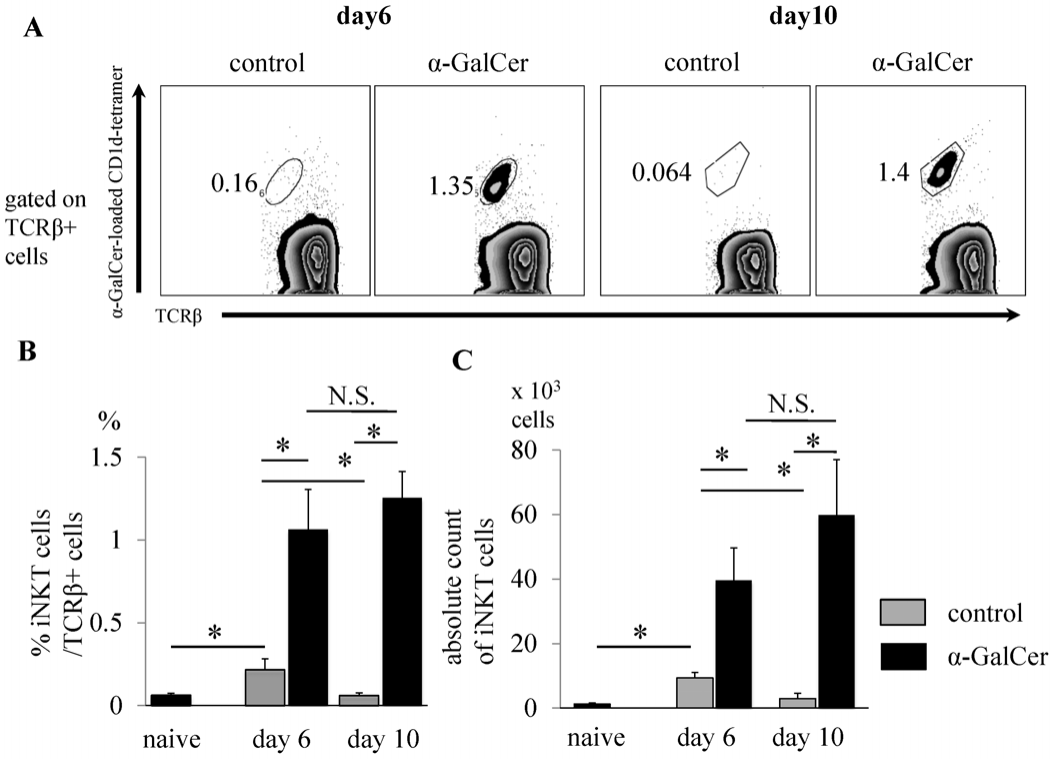
Expansion of iNKT cells in draining lymph nodes of α-GalCer-treated mice. Mice were immunized with GPI peptide and then treated with either DMSO or α-GalCer and euthanized on day 6 or 10. The dLNs were obtained and examined for the presence of iNKT cells by FCM (Data are mean±SD. n = 4–5. Data are representative results of two experiments). (**A**) iNKT cells were detected as TCRβ and α-GalCer-loaded CD1d-tetramer double positive cells. (**B**) Proportion of iNKT cells among TCRβ+ αβT cells, and (**C**) absolute number of iNKT cells in dLNs of naive or immunized mice. *p<0.05 by Man-Whitney analysis.

### Reagents

α-GalCer was purchased from Funakoshi (Tokyo, Japan). The stock solution of α-GalCer was dissolved in 100% dimethyl sulfoxide (DMSO) at 1 mg/ml and diluted in phosphate-buffered saline (PBS) just before injection. The following monoclonal antibodies (mAbs) were used for flow cytometric analysis: fluorescein isothiocyanate (FITC)-conjugated anti-CD4 (clone: RM4–5; Biolegend, San Diego, CA), alexa 488-conjugated foxp3 (clone: MF-14; Biolegend), phycoerythin-cyanine- 5 (PE/Cy5)-conjugated TCRβ (clone: H57–597; Biolegend), PE/Cy5-conjugated CD3 (clone; 145–2C11; Biolegend), allophycocyanin (APC)-conjugated CD19 (clone: 6D5; Biolegend), APC-conjugated CD25 (clone: PC61; Biolegend), and PE-conjugated CD1d-tetramer (MBL International, Woburn, MA). For the loading of CD1d-tetramer, the stock solution of α-GalCer was dissolved in pyridine at 1 mg/ml. Prior to use, α-GalCer was diluted to 0.2 mg/ml with 0.9% NaCl and 0.5% polysorbate-20. PE-conjugated CD1d tetramer was loaded overnight with α-GalCer at a ratio of 20∶1 at room temperature. Recombinant human GPI was prepared as described previously. GPI peptide was synthesized with 90% purity by Invitrogen (Carlsbad, CA) as described previously [18].

### GPI Peptide-induced Arthritis and Treatment with Glycolipids

GPI peptide was dissolved in DMSO. Prior to use, GPI peptide in DMSO was dissolved in Ringer solution to appropriate volume. Mice were immunized intradermally with 10 µg of GPI peptide in complete Freund’s adjuvant (CFA) (Difco, Detroit, MI). GPI peptide and CFA were emulsified at a 1∶1 ratio (volume/volume). For induction of arthritis, 150 µl of the emulsion was injected intradermally into the base of the tail. On days 0 and 2 after the immunization, 200 ng of pertussis toxin (Sigma-Aldrich, St. Louis, MO) was injected into mice intraperitoneally to develop arthritis. Mice were then treated with DMSO (vehicle control of α-GalCer) or 2 µg of α-GalCer on day 0 (with GPI peptide and CFA) or day 10 (with CFA). All the α-GalCer treatments were single and intradermal injections. Arthritis was evaluated visually, and changes in each paw were scored on a scale of 0–3, as described previously [17].

**Figure 3:**
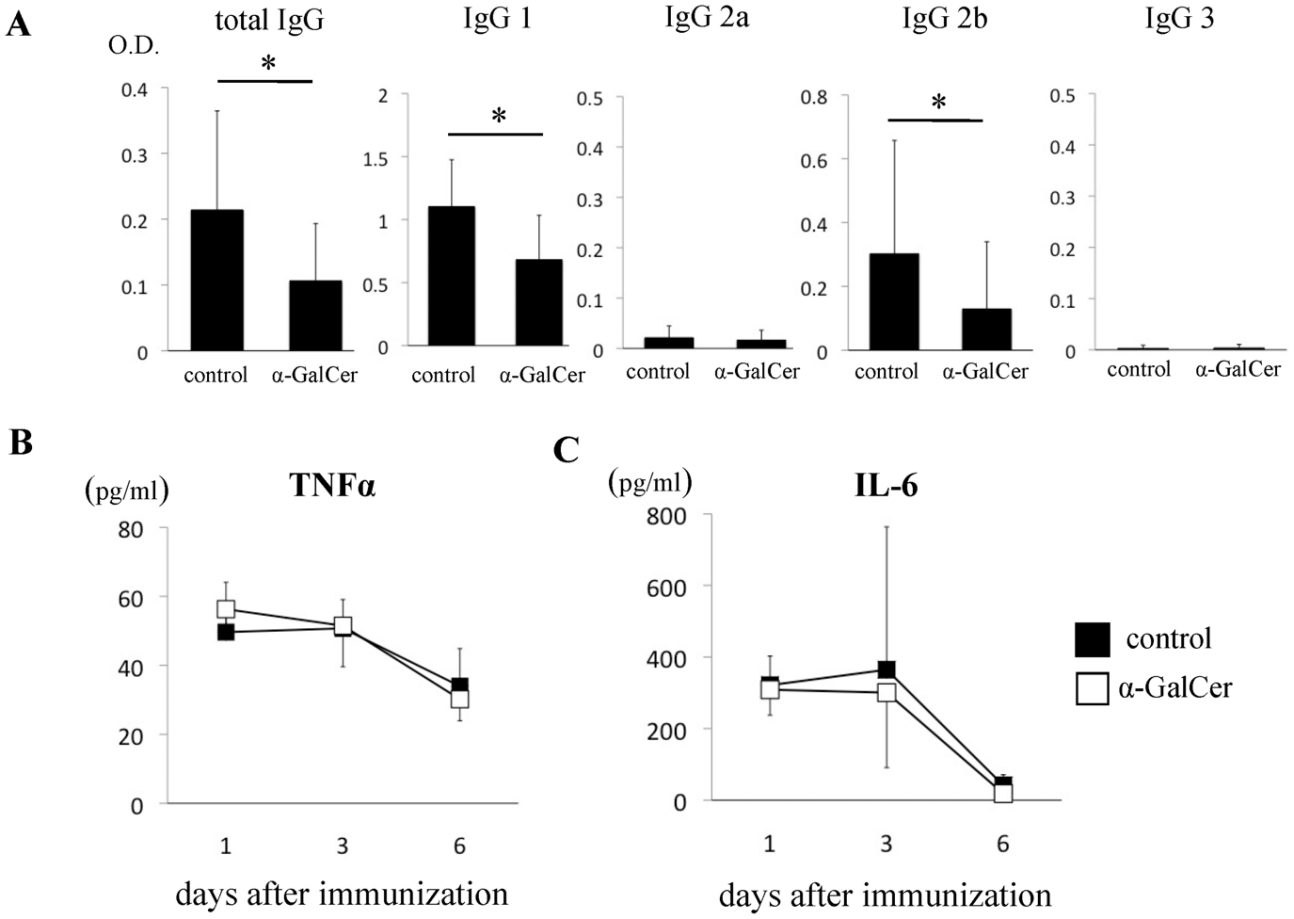
α-GalCer suppressed the production of autoantibodies against GPI, but not serum inflammatory cytokines. Mice were immunized with GPI peptide and treated with either DMSO or α-GalCer. Serum samples were obtained on days 1, 3, 6, and 28 after the immunization. (**A**) Levels of anti-recombinant human GPI antibody (total IgG, IgG1, IgG2a, IgG2b, IgG3) in sera obtained on day 28 were measured by ELISA (n = 11). Levels of (**B**) TNF-α and (**C**) IL-6 in the sera were measured by CBA (n = 3–5). Data are mean±SD. *p<0.05, by Man-Whitney analysis.

### Detection of Anti-GPI-antibody

Sera were obtained on day 28 and analyzed for the existence of anti-GPI antibody by enzyme-linked immunosorbent assay (ELISA). Sera were diluted 1∶1000 (for IgG) or 1∶100 (for IgG1, IgG2a, IgG2b, IgG3) in blocking solution (25% Block Ace (Dainippon Sumitomo Pharma, Osaka, Japan) in PBS). Then, 96-well plates were coated with 5 µg/ml of recombinant human GPI for 12 h at 4°C. After washing twice with washing buffer (0.05% Tween20 in PBS), the blocking solution was applied for 2 h at room temperature to block nonspecific binding. After 2 washes, 150 ml of diluted sera were added, and the plates were incubated for 2 h at room temperature. After three washes, horseradish peroxidase (HRP)-conjugated rabbit anti-mouse IgG (Dako, Glostrup, Denmark), IgG1 (Rockland, PA), IgG2a, IgG2b, (Zymed, San Francisco, CA), and IgG3 (Invitrogen) were added at a final concentration of 1∶1000 for 1 h at room temperature. After three washes, color was developed with TMB microwell peroxidase substrate (Funakoshi). The optical density was read at 450 nm using a microplate reader.

**Figure 4:**
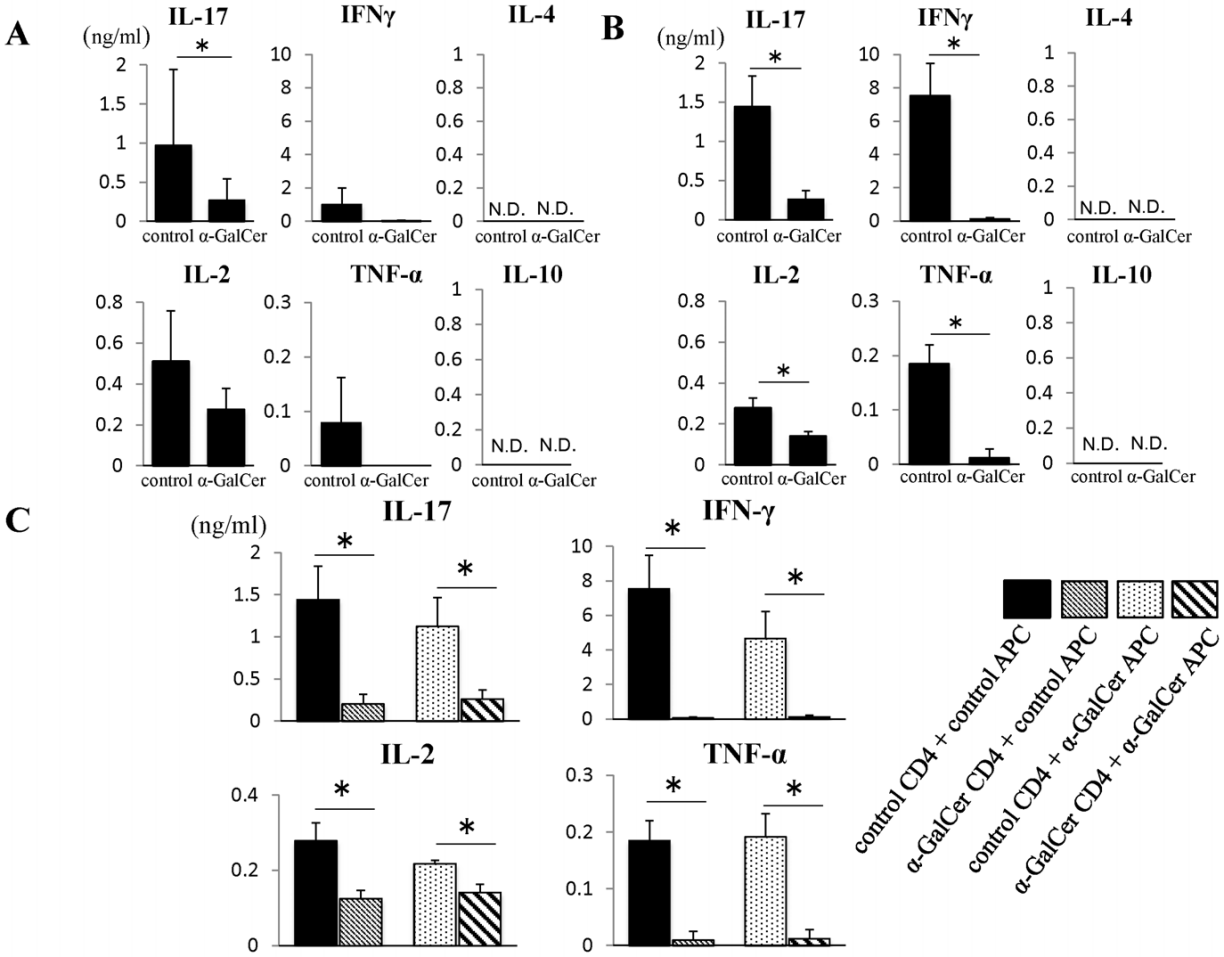
Suppression of antigen-specific CD4+ T cells by α-GalCer. (**A**)(**B**) Mice were immunized with GPI peptide and treated with either DMSO or α-GalCer. On day 6 (A) or day 10 (B), mice were euthanized and dLNs and spleens were harvested. CD4+ T cells were isolated from dLNs using MACS and cultured with mitomycin-treated splenocytes as antigen-presenting cells (APC). After 24-h culture, the levels of IL-17, IFN-γ, IL-2, TNF-α, IL-4 and IL-10 in the supernatant were measured by ELISA (n = 4). (**C**) Ten days after immunization, the mice were euthanized and dLNs and spleens were harvested. CD4+ T cells isolated from control or α-GalCer-treated mice were cultured with splenocytes (described as APC in the figure) from control or α-GalCer-treated mice. Cytokine levels in the culture supernatants were measured by ELISA (n = 4). Data are mean±SD. *p<0.05, by Man-Whitney analysis.

### Measurement of Serum Cytokines

Sera were obtained on days 1, 3, and 6 and analyzed for the levels of TNF-α and IL-6 by cytometric bead array (CBA) mouse inflammation kit (BD Biosciences, San Jose, CA) according to the instructions provided by the manufacturer.

### Assessment of Recall Response from Antigen-specific T cells

Mice were euthanized 6 or 10 days after immunization. The draining lymph nodes (dLN) and splenocytes were isolated as described previously [20]. Single-cell suspensions were prepared in Roswell Park Memorial Institute (RPMI) 1640 medium (Sigma-Aldrich) containing 10% fetal bovine serum (FBS), penicillin–streptomycin (100 U/ml), 10 mM HEPES buffer solution (Gibco BRL, Grand Island, NY), 0.1 mM MEM nonessential amino acids (Gibco), 1 mM sodium pyruvate (Gibco), and 5.5 mM 2-mercaptoethanol (2-ME). CD4+ T cells were isolated from dLN cells by magnetic-activated cell sorting (MACS, Miltenyi Biotec, Bergisch Gladbach, Germany). The purity (more than 95%) was confirmed by flow cytometry. Splenocytes were incubated with 50 µg/ml mitomycin C (MMC) (Sigma-Aldrich) for 30 min at 37°C in a water bath. Purified CD4+ T cells and MMC-treated splenocytes were co-cultured in the presence of 10 µM of GPI peptide at a ratio of 1∶1 for 24 h at 37°C under 5% CO_2_−95% air environment. In other experiments, MMC-treated non-T cells were used instead of splenocytes. The supernatants were assayed for IL-17, IFN-γ, IL-2, TNF-α, IL-4 and IL-10 by ELISA using Quantikine ELISA kit (R&D Systems, Minneapolis, MN).

**Figure 5:**
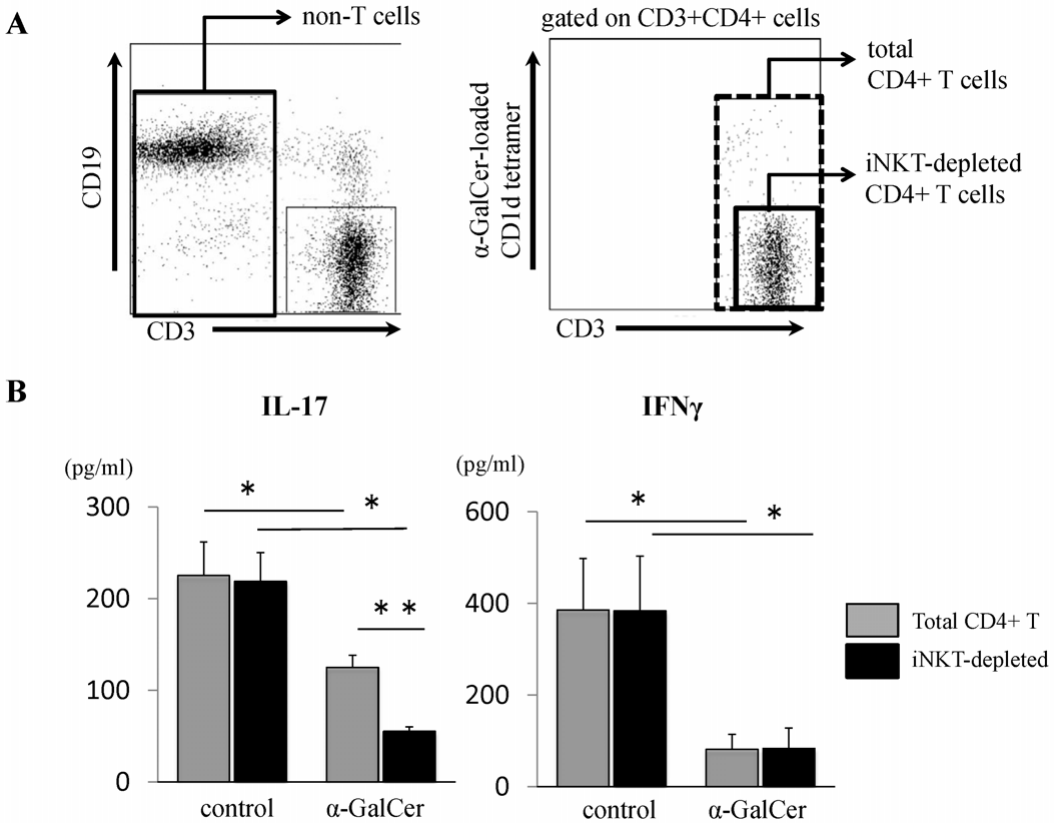
iNKT cells do not directly suppress antigen-specific CD4+ T cells *in vitro*. Mice were immunized with GPI peptide and treated with either DMSO or α-GalCer. Mice were euthanized on day 10 and dLNs were harvested. (**A**) Total CD4+T cells (CD3+ CD4+ cells) and NKT-depleted CD4+T cells (CD3+ CD4+ α-GalCer loaded CD1d-tetramer negative cells) were sorted by flow cytometry. These cells were cultured with sorted MMC-treated CD3-negative cells as APC in the presence of 10 µM of GPI peptide for 24 h. (**B**) Supernatants were collected and subjects to quantitative analysis of IL-17 and IFN-γ levels by ELISA. Data are mean±SD. (n = 4–5). *p<0.05, **p<0.01, by Man-Whitney analysis.

### Statistical Analysis

Values are expressed as mean ± SD. Differences between groups were examined for statistical significance using the Man-Whitney’s U test. Probability values less than 0.05 were considered significant. All analyses were conducted using The Statistical Package for Social Sciences software version 19 (SPSS Inc., Chicago, IL).

## Results

### α-GalCer reduces the Severity of GPI Peptide-induced Arthritis

We first evaluated whether α-GalCer has a protective effect on GPI peptide-induced arthritis. Mice were intradermally immunized with GPI peptide and treated with either DMSO or α-GalCer. The severity of arthritis was evaluated by measuring the clinical score and ankle thickness. The arthritis score on day 14 was significantly lower in α-GalCer-treated mice (3.8±2.1) than in control mice (10.7±1.5, P = 0.004, **Fig. 1A and B**). Furthermore, the ankle thickness was significantly less in α-GalCer-treated mice (3.34±0.2 mm) than in control mice (3.98±0.26 mm, P = 0.008, **Fig. 1A and C**). To elucidate whether α-GalCer has protective effect against arthritis when administered after the initiation of arthritis, GPI peptide-immunized mice were intradermally administered with DMSO or α-GalCer on day 10. However, there was no significant difference between the groups (**Fig. S1**). The arthritis scores on day 14 were 9.6±1.5 in control mice, and 8.6±1.1 in α-GalCer-treated mice.

**Figure 6:**
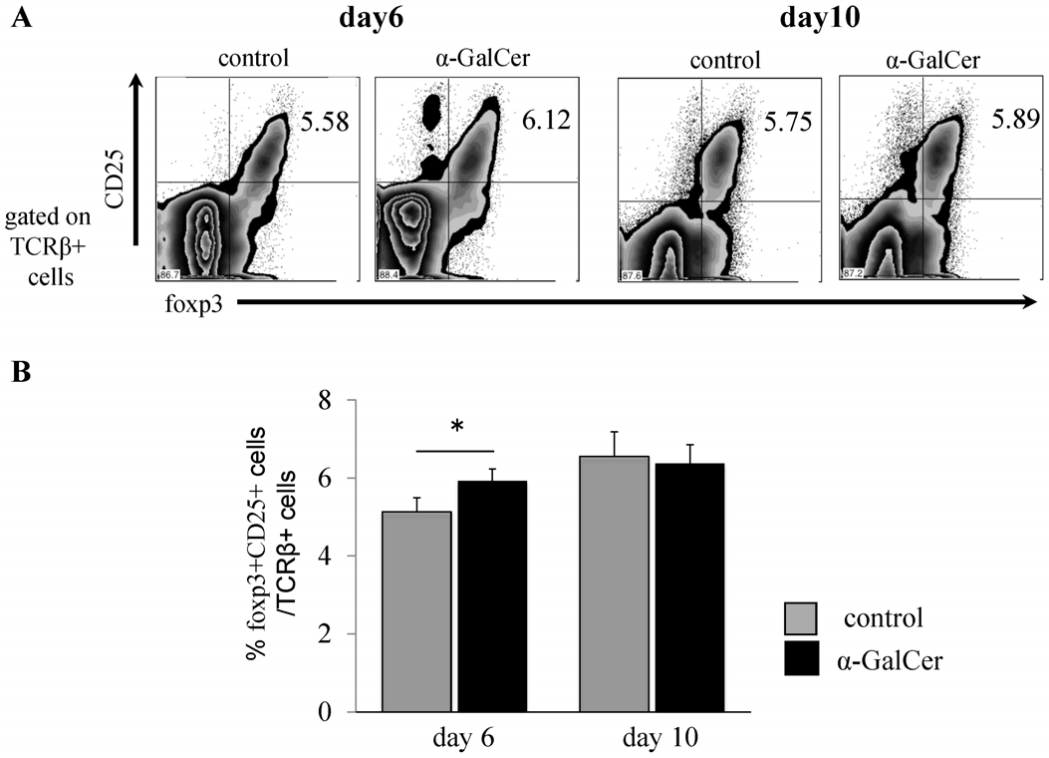
α-GalCer does not induce expansion of CD25+foxp3+ regulatory T (Treg) cells. Mice were immunized with GPI peptide and treated with either DMSO or α-GalCer, then euthanized on day 6 or 10 and dLN cells were harvested. (**A**) Foxp3+ T reg cells were detected as TCRβ, foxp3- and CD25-positive cells by flow cytometry. (**B**) Proportion of foxp3+ CD25+ cells among TCRβ+ αβ T cells. Data are mean±SD. *p<0.05, by Man-Whitney analysis.

### α-GalCer Induces iNKT Cell Expansion in Draining Lymph Nodes

GPI peptide-immunized mice treated with DMSO or α-GalCer were euthanized on day 6 or 10, and the dLNs were isolated and prepared for examination of iNKT cells. iNKT cells were identified as α-GalCer-loaded CD1d-tetramer and TCRβ double positive cells **(** **Fig. 2A** **)**. Naive mice were also investigated for the presence of iNKT cells in the inguinal lymph nodes. The proportion and absolute number of iNKT cells in the dLNs on day 6 were significantly higher in the control peptide-treated mice (0.22±0.067%, 9.4±1.7×10^3^ cells, respectively) than in naive mice (0.081±0.018%, 2.6±1.7×10^3^ cells, respectively) (**Fig. 2B and C**). Surprisingly, the proportion and absolute number of iNKT cells in the dLNs were significantly higher in α-GalCer-treated mice than in control mice both on day 6 (1.1±0.24%; P = 0.021, 40±10×10^3^ cells; P = 0.021, respectively) and day 10 (α-GalCer-treated mice: 1.26±0.15% and 60±17×10^3^ cells, control: 0.061±0.017%, 3.0±1.7×10^3^ cells, P = 0.021, respectively (**Fig. 2B and C**).

### Effects of α-GalCer on GPI-specific Antibody Production

To investigate the effects of α-GalCer on GPI-specific antibody production, serum samples were obtained from control and α-GalCer-treated mice on day 28 and assayed for the levels of anti-GPI antibodies by ELISA. Since low levels of anti-GPI antibodies, especially IgG subtypes, could help identify the type of T helper cells that mediate the effect of α-GalCer, we also analyzed IgG subclasses (IgG1, 2a, 2b, 3). Administration of α-GalCer significantly reduced the production of anti-GPI antibodies of IgG, IgG1, and IgG2b compared with the control mice (IgG: 0.11±0.087 *vs* 0.21±0.15; P = 0.029, IgG1∶0.68±0.35 *vs* 1.1±0.37; P = 0.014, IgG2b: 0.13±0.21 *vs* 0.30±0.35; P = 0.049, respectively) **(** **Fig. 3A** **)**. These results suggest that α-GalCer suppressed antigen-specific responses independently of subsets of T helper cells, e.g., Th1 or Th2 cells.

We also analyzed the effects of α-GalCer on inflammatory cytokines on days 1, 3, and 6. Treatment with α-GalCer did not alter the serum levels of TNF-α (control: 49±14, α-GalCer: 56±5.8 pg/ml) and IL-6 (control: 321±81, α-GalCer: 309±71 pg/ml) measured on day 1 after immunization **(** **Fig. 3B and C** **)**. The levels of these cytokines decreased significantly in both the DMSO- and α-GalCer-treated mice at day 6, but not at day 3 **(** **Fig. 3B and C** **)**.

### Effects of α-GalCer on GPI Peptide-specific Recall Response of CD4 T cells

The fact that GPI peptide-induced arthritis is T cell dependent, especially IL-17-producing CD4+ T cells, we also examined the effects of α-GalCer on antigen-specific CD4+ T cells. To evaluate antigen-recall response of CD4+ T cells, immunized mice were euthanized on day 6 or day 10. The isolated CD4+ T cells were co-cultured with MMC-treated whole splenocytes as APC in the presence of GPI peptide. Then, the levels of IL-17, IFN-γ, IL-2, TNF-α, IL-4, and IL-10 in the culture supernatants were measured by ELISA. On day 6, only IL-17 production was significantly suppressed in α-GalCer-treated mice (control: 1588±972 pg/ml, α-GalCer: 562±272 pg/ml; P = 0.028, **Fig. 4A**). On day 10, levels of IL-17, IFN-γ, IL-2, and TNF-α were significantly lower in α-GalCer-treated mice than the control (IL-17∶261±110 *vs* 1443±393 pg/ml; P = 0.014, IFN-γ: 132±82 *vs* 7536±1936 pg/ml; P = 0.014, IL-2∶141±21.5 *vs* 279±47 pg/ml; P = 0.014, TNF-α: 11.9±16.3 *vs* 185±35 pg/ml; P = 0.012, respectively, **Fig. 4B**). IL-4 and IL-10 were not detected in this assay.

To determine the roles of CD4+ T cells and APCs in the above suppressive effect, CD4+ cells of the DMSO- and α-GalCer-treated mice were cultured with APCs from control or α-GalCer-treated mice in the presence of GPI peptide, then analyzed for cytokine production in response to antigen re-stimulation. Impaired recall response was noted when CD4+ T cells from α-GalCer-treated mice were cultured with APCs from control mice (IL-17∶1443±393 *vs* 203±113 pg/ml; P = 0.014, IFN-γ: 7536±1936 *vs* 66±60 pg/ml; P = 0.028, IL-2∶279±47 *vs* 125±21.5 pg/ml; P = 0.025, TNF-α: 185±35 *vs* 11±15 pg/ml; P = 0.022 in control CD4+control APC and α-GalCer CD4+control APC group, respectively) (**Fig. 4C**). On the other hand, no such impairment of recall response was noted when CD4+ T cells from control mice were cultured with APCs from α-GalCer-treated mice (IL-17∶1124±342 *vs* 261±110 pg/ml; P = 0.025, IFN-γ: 4674±1550 *vs* 132±82 pg/ml; P = 0.025, IL-2∶219±9.2 *vs* 141±22 pg/ml; P = 0.025, TNF-α: 192±40 *vs* 12±16 pg/ml; P = 0.022 in control CD4+α-GalCer APC and α-GalCer CD4+α-GalCer APC group, respectively) **(** **Fig. 4C** **)**. These results suggest impairment of antigen-specific response in α-GalCer-treated mice and that CD4+ T cells are responsible for the impairment.

### Recall Response of iNKT-depleted CD4+ T cells

Because dLN CD4+ T cells contain approximately 1% of iNKT cells, we questioned whether the expanding iNKT cells among dLN CD4+ T cells had direct suppressive effects on antigen-specific CD4+ T cells. To answer the question, we evaluated the recall response of iNKT-depleted CD4+ T cells. iNKT-depleted CD4+ T cells, total CD4+ T cells, and CD3- non-T cells were sorted from dLN cells of immunized mice on day 10 **(** **Fig. 5A** **)**. These CD4+ T cells were cultured with non-T cells in the presence of GPI peptide. The production of IL-17 and IFN-γ was suppressed from total CD4+ cells of α-GalCer-treated mice (IL-17: control: 225±37, α-GalCer: 125±13 pg/ml; P = 0.014, IFN-γ: control: 386±112, α-GalCer: 81±33 pg/ml; P = 0.014) **(** **Fig. 5B** **)**. The production of these cytokine was still suppressed in iNKT-depleted CD4+ T cells from α-GalCer-treated mice (IL-17: control: 219±31, α-GalCer: 55±4.9 pg/ml; P = 0.014, IFN-γ: control: 383±120, α-GalCer: 83±44 pg/ml; P = 0.037) **(** **Fig. 5B** **)**. IL-17 production by GPI reactive T cells was significantly reduced under iNKT -depleted condition (total CD4+ T cells: 125±13, iNKT-depleted CD4+T cells: 55±4.9 pg/ml, P = 0.009) **(** **Fig. 5B** **)**. These results suggest that expanded iNKT cells did not have direct suppressive effects on antigen-specific CD4+ T cells in this assay.

### Analysis of foxp3^+^ Regulatory T cells

To determine whether induction of foxp3+ regulatory T cells mediates the suppressive effect of α-GalCer on antigen-specific CD4+ cells, we examined dLN cells of immunized mice on days 6 and 10 for the presence of foxp3+ CD25+ TCRβ+ T cells by FCM **(** **Fig. 6A and B** **)**. The proportion of foxp3+ T reg cells was significantly higher in α-GalCer-treated mice than in control mice on day 6 (control: 5.1±0.35%, α-GalCer: 5.93±0.30%, P = 0.043) but not on day10 (control: 6.6±0.62%, α-GalCer: 6.4±0.48%) **(** **Fig. 6B** **)**. However, based on the small difference, we concluded that the difference was not the reason for the potent suppressive effect of α-GalCer on pathogenic CD4+ T cells.

## Discussion

GPI-induced arthritis is a newer and closer model of human RA with regard to its dependency on CD4+ T cells and reactivity to biological treatments such as blockade of IL-6 and TNF-α, which are well known to be effective in human RA. Because of its dependency on T cells, the dominant T cell epitope, which is just 15 mer peptide, can induce arthritis that resembles GPI-induced arthritis. Although accumulating evidence point to the protective role of iNKT cells against autoimmune demyelination [6], [7], it is still unclear how iNKT cells work and control autoimmune arthritis, particularly through their exogenous-ligand activation. Although iNKT cells are known to promote autoimmune arthritis such as CIA, collagen-antibody induced arthritis, and K/BxN serum transfer arthritis [21], they might work differently in a ligand-specific manner. The present study was designed to explore the role of α-GalCer in GPI peptide-induced arthritis.

The results demonstrated that α-GalCer potently suppressed the severity of GPI-peptide induced arthritis. Although the severity of arthritis on day 10 was not different between the control and α-GalCer-treated mice, the former group developed more severe arthritis up to day 14 whereas α-GalCer-treated mice had a milder form of arthritis. Intradermal administration of α-GalCer with GPI peptide is delivered into dLNs by dendritic cells (DCs) residing in the skin and presented to iNKT cells by dLN DCs [22]. These facts mean that iNKT cell proliferated in the dLNs prior to the onset of arthritis. Hence, we speculated that α-GalCer-activated iNKT cells regulated GPI-specific immune response through acquisition of regulatory phenotypes or induction of regulatory cells, such as T reg cells, as reported in previous studies [11], [12]. However, α-GalCer-activated iNKT cells neither induced foxp3+ CD25+ regulatory T cells nor had direct regulatory effects on antigen-specific CD4+ T cells *in vitro*. Further studies of other T reg cells might be necessary. Although iNKT cells did not have direct suppressive effects on differentiated effector T cells, they might affect helper T cell differentiation *in vivo* in the induction phase of arthritis. **Oh et al.** reported iNKT cells suppress pathogenic Th17 cells through iNKT cell - T cell interaction without cytokines in EAE [23]. It is possible that expansion of iNKT cells in dLNs enhances the suppression of differentiation of naive T helper cells into pathogenic effector T cells.

Our previous studies [18], [24] demonstrated that immunization using GPI peptide induced IL-17-producing CD4+ T cells while blockade of IL-17 resulted in amelioration of arthritis, suggesting that IL-17-producing CD4+ T cells play a pathogenic role in GPI peptide-induced arthritis. Since mice deficient in IFN-γ receptor were more resistant to GPI-induced arthritis, IFN-γ may also play a pathogenic role in GPI-induced arthritis [25]. In the present study, peptide-specific responses were significantly suppressed in α-GalCer-treated mice on day 10. These results suggest that the milder form of arthritis in α-GalCer-treated mice could have been mediated, at least in part, through the suppression of IL-17- and IFN-γ-producing T cells. **Kaieda et al.** showed that SGL-S23, an analog of α-GalCer, had suppressive effect on K/BxN serum transfer arthritis through IFN-γ [26]. We previously showed that α-carba-GalCer, an analog of α-GalCer, suppressed the severity of CIA through the induction of Th1-biased differentiation of CD4+ T cells [20]. In contrast to these studies, α-GalCer suppressed both Th1 and Th17 CD4+ T cells in GPI peptide-induced arthritis. **Coppieters et al.** reported the proliferation of IL-10-producing T cells in α-GalCer-treated mice during the course of CIA [9], and **Miellot et al.** showed that α-GalCer ameliorated CIA through the induction of IL-10-producing antigen-specific CD4+ T cells and that the protective effect was canceled by blockade of IL-10 using anti-IL-10 antibody [8]. **Chiba et al.** reported the Th2-polarizing glycolipid ligand of iNKT cells, OCH, had a potent therapeutic effect against CIA [27]. However, in our study, the protective effect of α-GalCer against arthritis was not due to the induction of antigen-specific IL-10 producing cells. Administration of α-GalCer reduced anti-GPI antibody levels independent of T helper cell subclasses. These results suggest that α-GalCer suppressed the whole antigen-specific CD4+ T cells independent of their subsets (e.g., as Th1, Th2, and Th17). In contrast to the clear effect of prophylactic administration, therapeutic α-GalCer administration on day 10 had no effect on the severity of arthritis. **Kim et al.** reported activation of iNKT cells by α-GalCer had no effect on K/BxN serum transfer arthritis in which T and B cells are not required [28], [29]. These facts also suggest that the suppressive effect of α-GalCer is mediated by regulation of antigen-specific T cell development.

In conclusion, the present study demonstrated that α-GalCer significantly suppressed GPI peptide-induced arthritis through the suppression of antigen-specific CD4+ T cells. Further studies are needed to determine the mechanism through which α-GalCer-activated iNKT cells suppress the induction of antigen-specific CD4+ T cells. Such studies could advance the development of antigen-specific vaccination therapy against human RA.

## Supporting Information

Figure S1 **Therapeutic administration of α-GalCer had no effect on the severity of GPI peptide-induced arthritis.** DBA1 mice were immunized with GPI peptide and then treated with either DMSO (as a vehicle control) or α-GalCer on day 10 followed by clinical assessment of arthritis. (n = 5). Clinical score is shown in the figure. Arrow indicates α-GalCer administration.
(TIFF)
