# Supplementary figures and images for: Activation of Invariant NKT Cells with Glycolipid Ligand α-Galactosylceramide Ameliorates Glucose-6-Phosphate Isomerase Peptide-Induced Arthritis
Source: PLoS One. 2012 Dec 12;7(12):e51215. doi: 10.1371/journal.pone.0051215 (PMC3520964; doi:10.1371/journal.pone.0051215)

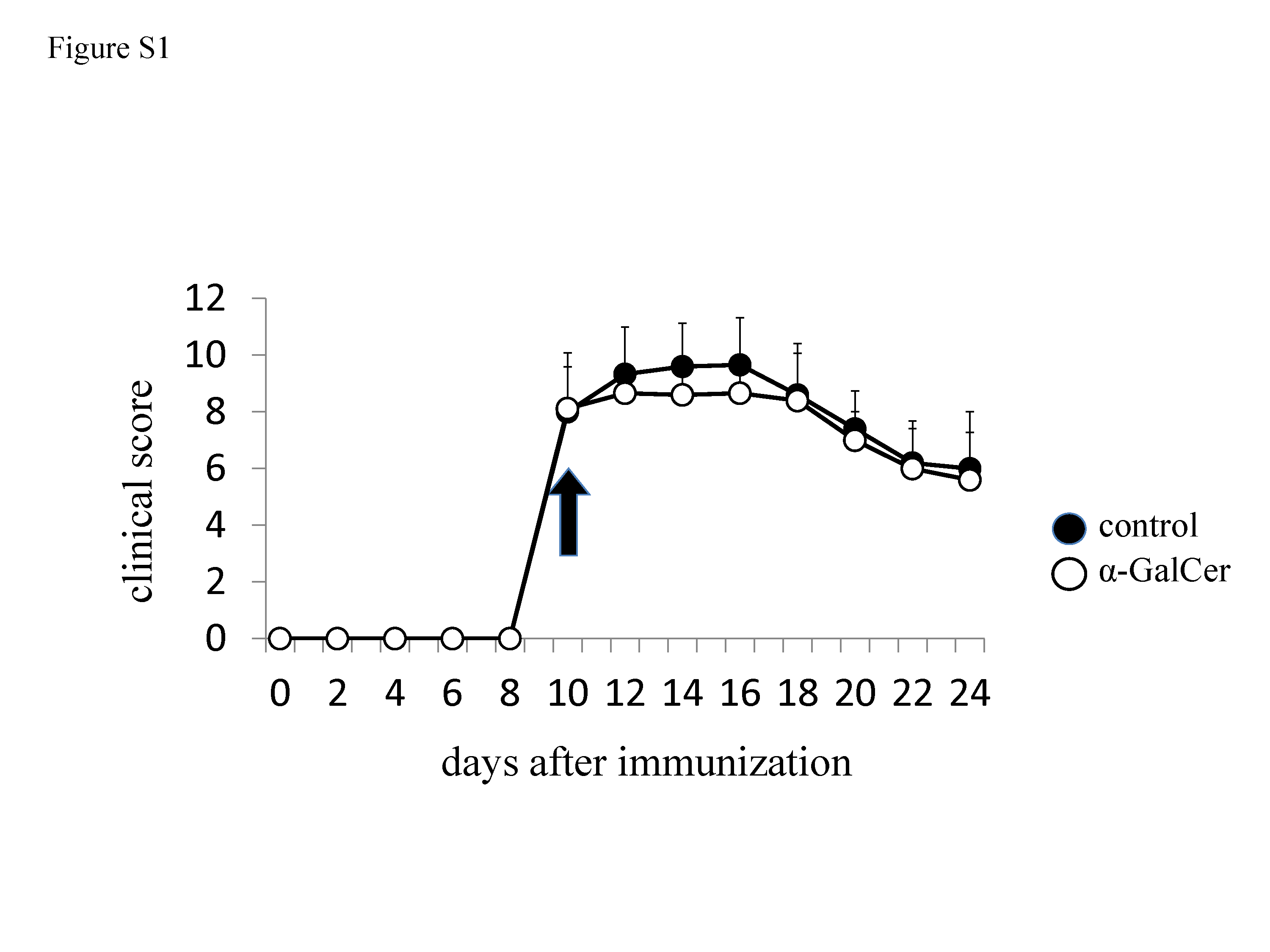

Supplement: Figure S1 — Therapeutic administration of α-GalCer had no effect on the severity of GPI peptide-induced arthritis. DBA1 mice were immunized with GPI peptide and then treated with either DMSO (as a vehicle control) or α-GalCer on day 10 followed by clinical assessment of arthritis. (n = 5). Clinical score is shown in the figure. Arrow indicates α-GalCer administration. (TIFF) [file pone.0051215.s001.tiff]
